# Successful laparoscopic management of combined traumatic diaphragmatic rupture and abdominal wall hernia: a case report

**DOI:** 10.1186/s13256-015-0780-8

**Published:** 2016-01-18

**Authors:** Sze Li Siow, Chee Ming Wong, Mark Hardin, Mushtaq Sohail

**Affiliations:** 1 Department of Surgery, Sarawak General Hospital, Jalan Hospital, 93586 Kuching, Sarawak Malaysia; 2 Department of Surgery, Faculty of Medicine and Health Sciences, Universiti Malaysia Sarawak, 94300 Kota Samarahan, Sarawak Malaysia

**Keywords:** Motor vehicle collision, Traumatic diaphragmatic rupture, Traumatic abdominal wall hernia, Blunt trauma, Mesh repair

## Abstract

**Background:**

Traumatic diaphragmatic rupture and traumatic abdominal wall hernia are two well-described but rare clinical entities associated with blunt thoracoabdominal injuries. To the best of our knowledge, the combination of these two clinical entities as a result of a motor vehicle accident has not been previously reported.

**Case presentation:**

A 32-year-old Indian man was brought to our emergency department after being involved in a road traffic accident. He described a temporary loss of consciousness and had multiple tender bruises at his right upper anterior abdominal wall and left lumbar region. An initial examination revealed blood pressure of 99/63 mmHg, heart rate of 107 beats/minute, and oxygen saturation of 93 % on room air. His clinical parameters stabilized after initial resuscitation. A computed tomographic scan revealed a rupture of the left diaphragm as well as extensive disruptions of the left upper anterior abdominal wall. We performed exploratory laparoscopic surgery with the intention of primary repair. The diaphragmatic and abdominal wall defect was primarily closed, followed by reinforcement with PROLENE onlay mesh. The patient’s postoperative recovery was complicated by infected hematomas over both flanks that were managed with ultrasound-guided percutaneous drainage. He was discharged well despite a prolonged hospital stay.

**Conclusions:**

We present a complex form of injuries managed successfully via a laparoscopic approach. Meticulous attention to potential complications in both the acute and convalescent phases is important for achieving a successful outcome following surgery.

## Background

Traumatic diaphragmatic rupture (TDR) and traumatic abdominal wall hernia (TAWH) are rare, albeit well-documented, clinical entities associated with blunt thoracic and abdominal injuries. The mechanism of injury for TDR and TAWH is similar and related to either blunt or penetrating trauma. TDRs have been reported either as an isolated injury or in association with other abdominal injuries. However, to the best of our knowledge, the combination of TDR and TAWH as a result of a motor vehicle accident has not been reported to date. We present such a case and describe the surgical approach we used and the complications encountered during the recovery phase. The term *rupture* is synonymous with tear and is defined as a break or disruption of tissue. *Defect* is defined as an abnormal opening in the anatomical structure. Sarawak General Hospital, with which the authors are affiliated, is the main tertiary and referral hospital in the state of Sarawak, Malaysia. The hospital manages around 10,000 cases of trauma annually.

## Case presentation

**Fig. 1:**
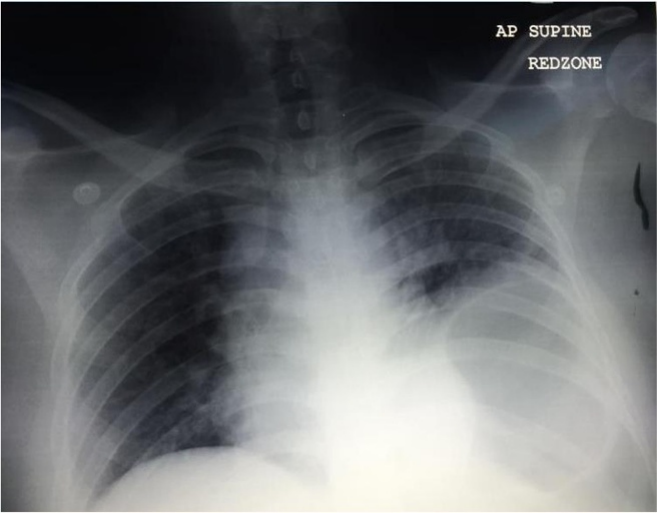
Chest x-ray showing a raised left hemidiaphragm with the presence of stomach shadow in the lower half of the left hemithorax

**Fig. 2:**
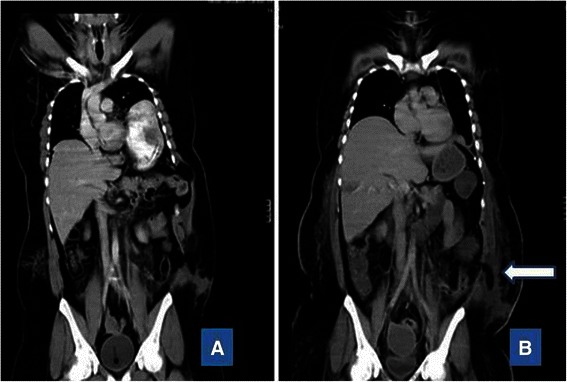
**a** Preoperative computed tomographic scan showing herniation of the patient’s stomach into the left thorax and of the small bowels into an abdominal wall defect. **b** Computed tomographic scan of the patient’s abdomen obtained at 2 weeks after the accident showing fat stranding and presence of air locules suggestive of infection of abdominal wall collection (*arrow*)

A 32-year-old Indian man was brought to our emergency department after being involved in a road traffic accident. His current medical history included obesity, with a body mass index of 38 kg/m^2^, and treated hypertension. He was in the front seat of a passenger car that was involved in a head-on collision with another car. He described a temporary loss of consciousness and had multiple tender bruises at his right upper anterior abdominal wall and left lumbar region. An initial examination revealed blood pressure of 99/63 mmHg, heart rate of 107 beats/minute, and oxygen saturation of 93 % in room air. The patient’s Injury Severity Score (ISS) was 17 (Table 1). His clinical parameters stabilized after initial fluid resuscitation and supplemental oxygen. Arterial blood gas analysis (on nasal prong O_2_ at 2 L/minute) showed a pH of 7.36, partial pressure of oxygen of 90.6 mmHg, oxyhemoglobin saturation of 96.5 %, partial pressure of carbon dioxide of 42.5 mmHg, bicarbonate ion of 23.3 mmol/L, and base excess of −2.2 mmol/L. The initial chest radiograph demonstrated an elevated left hemidiaphragm with the presence of a stomach gas shadow in the lower half of the hemithorax (Fig. 1). Computed tomography (CT) of the patient’s abdomen showed a large posterolateral left diaphragmatic defect with herniation of the stomach into the left thorax, together with herniation of the small bowel through the left eighth intercostal space and an adjacent rent through the left transversus abdominis muscle (Fig. 2a). The patient was informed of the surgical options of both laparoscopic and laparotomy approaches. He fully understood and chose to have laparoscopic exploration with the intention of primary repair.

**Table 1:** Injury Severity Score

Region	Injury	AIS	AIS^2^
Head and neck	Cerebral concussion	1	1
Face	No injury	0	0
Chest	1. Avulsion of chest wall tissues with underlying rib fractures	4	
	2. Laceration of diaphragm >10 cm with tissue loss <25 cm^2^	3	16
Abdomen	No injury	0	0
Extremity	No injury	0	0
External	No injury	0	0
ISS = 17			

*AIS* Abbreviated Injury Scale, *AIS*^2^ Abbreviated Injury Scale Square, *ISS* Injury Severity Score

**Fig. 3:**
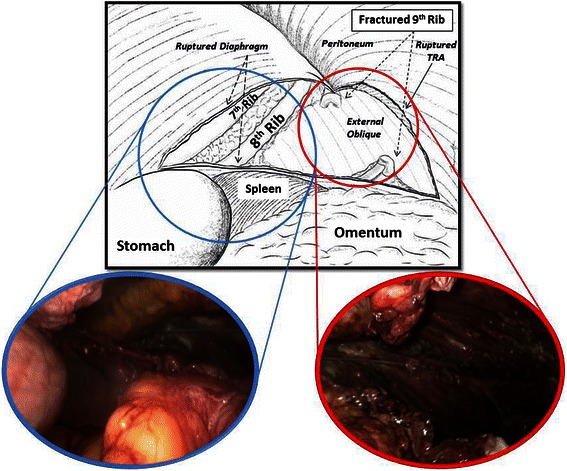
Schematic illustration with intraoperative view of traumatic diaphragmatic rupture and traumatic abdominal wall hernia. *TRA* transversus abdominis muscle

Perioperatively, we found a 10-cm-long radial tear in the posterolateral aspect of the diaphragm, which extended to the left anterior abdominal wall with complete disruption of all muscular layers (Fig. 3). The patient’s stomach and omentum had herniated through the diaphragmatic defect. There was an associated left ninth rib fracture with torn intercostal muscles between the eighth and ninth ribs (Fig. 3). The patient’s small bowel had herniated into the abdominal wall defect. After successful reduction of the hernia contents, the diaphragmatic defect and the peritoneum over the abdominal wall defect were closed primarily with a running, nonabsorbable 2−0 polyester suture. Due to the size of the diaphragmatic and abdominal wall defect, we decided to reinforce the repair using a nonabsorbable polypropylene composite mesh (PROCEED® Surgical Mesh; Ethicon Endo-Surgery, Norderstedt, Germany) secured to the diaphragm and peritoneum with tackers (ProTack^TM^ 5 mm; Covidien, Mansfield, MA, USA) circumferentially overlapping the margins of the primary repair by 5 cm. A chest tube was inserted into the left pleural space, and a vacuum drain (Redivac; Primed, Halberstadt, Germany) was placed into the abdominal wall defect through the left midclavicular port at the end of the surgery.

**Fig. 4:**
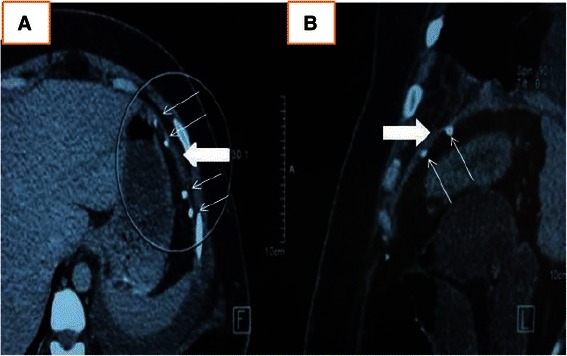
Axial (**a**) and sagittal (**b**) computed tomographic images showing continuity of the left hemidiaphragm (highlighted within the *circle*) reinforced with mesh (*thick arrows*) and ProTack (*narrow arrows*)

The patient was extubated on postoperative day 1 and spent another 4 days in the intensive care unit before he was transferred to the open ward. His chest tube was removed after 3 days of minimal drainage. The left abdominal wall defect Redivac drain was kept in place longer, as there was a significant amount of hemoserous drainage. The patient was progressing well until postoperative day 7, when he experienced severe lower abdominal pain around his waist circumference. A repeat CT scan of his abdomen showed fluid collection in both the right and left lower anterior abdominal walls, with associated left pleural effusion and bibasal and biapical consolidation of the lungs. The Redivac drain was maintained, and he was managed expectantly with intravenous antibiotics and analgesics. Unfortunately, 2 weeks after the accident, the patient developed temperature spikes with purulent discharge from the drain. The initial concern was that the mesh had become infected with possible empyema thoracis. However, there was no evidence on repeated CT scanning of the thorax and abdomen to suggest such findings. In addition, CT showed continuity of the left hemidiaphragm reinforced with mesh and ProTack (Fig. 4). Nevertheless, there was extensive fat stranding in the previous abdominal wall fluid collections, along with presence of some air locules, suggestive of superimposed infection (Fig. 2b). Pus culture from the abdominal wall drain isolated *Hafnia alvei* sensitive to amoxicillin/clavulanic acid, and the patient’s antibiotic was downgraded from the initial intravenous meropenem 500 mg three times daily to intravenous amoxicillin/clavulanic acid 1.2 g three times daily for 1 week. The Redivac drain was removed on postoperative day 23, when the purulent discharge had become minimal. A few days after removal of the drain, the patient developed spikes of temperature. Ultrasound (US) examination of the abdomen showed two collections: one at the right anterior abdominal wall, measuring 2.8 × 12.1 cm and another at the left anterior abdominal wall measuring 5.7 × 14.6 cm. US-guided percutaneous drainage was performed with insertion of two 8-French pigtail catheters. The aspirated pus grew *Yokenella regensburgei*, for which the patient was treated with appropriate antibiotics. The drainage gradually settled over the next 2 weeks, with repeat abdominal US showing resolution of the collection at the right anterior abdominal wall and a small residual collection at the left. He was discharged from the hospital after 42 days. At 6 months postoperatively, the patient was well with no evidence of abdominal wall hernia.

## Discussion

TDR with TAWH is a rare combination of injuries occurring due to a motor vehicle accident. To date, there has been no report about such a case in the literature. To the best of our knowledge, we present the first case of combined diaphragmatic and abdominal wall rupture secondary to trauma. The isolated injury of either condition is considered a marker of severe injury reflected by a high ISS [1–3]. Therefore, the combined injury is a marker of greater severity of injury. The mechanism of injury might be the consequence of (1) a direct blow to the thoracoabdominal wall causing an acute rise in intraabdominal pressure (in the case of TDR and TAWH) and a tangential force to the abdominal wall producing shearing stresses to the underlying muscles and fascia (in the case of TAWH), (2) deceleration injury accentuated by the lap-and-shoulder safety belt creating a pressure gradient between the pleural and peritoneal cavities (in the case of TDR) and across the abdominal wall (in the case of TAWH), and (3) the inherent weakness of the diaphragm at its posterolateral region, where it inserts into the chest wall (in the case of TDR). Our patient’s case is an example of frontal ipsilateral impact (as a front seat passenger) resulting in left-sided thoracoabdominal and diaphragmatic injuries. The combined TDR and TAWH with intraabdominal bowel herniation seen in this case is an example of deceleration injury. The fractured ninth rib where the diaphragm inserts may have aggravated the extent of both injuries.

The isolated incidences of TDR and TAWH and following blunt trauma are reported to be 0.2–1.9 % [2–4] and 0.9–17 % [5–7], respectively. However, the true incidence may be underestimated because of under- or misdiagnosis. The most common mechanism of injury leading to TDR and TAWH is a motor vehicle accident [1, 2, 5, 7, 8], with injury sustained following high-energy transfer. The other mechanism of injury described in TAWH is low-energy injury caused by a small blunt object, resulting in small defects [9]. The best example is the handlebar hernia, first described by Dimyan *et al.* in 1980 [10]. Most of TDR occurs on the left side of the diaphragm [2, 11]. The disparity in the location of the rupture could be explained by several factors [8, 12], including (1) the anatomical protection provided by the liver, resulting in greater force required to cause a rupture of right hemidiaphragm; and (2) the left hemidiaphragm being congenitally weaker than the right as a result of weakness in various points of diaphragmatic embryological fusion. Several types of diaphragmatic tears have been described, which include radial (most common), transverse, central, and peripheral tears [12]. The radial tear, as in our patient, is an example of a tear that occurs at the posterolateral aspect of the muscle, where the diaphragm is avulsed from its point of attachment. However, the location of TAWH varies according to the mechanism of injury. The most common locations are the lumbar region and the lower abdomen, corresponding to areas of relative anatomic weakness and transmission of force from a lap belt during abrupt deceleration [5].

Chest radiography and CT are two important diagnostic tools for trauma assessment. A chest radiograph alone is not sufficient to exclude TDR. A high index of suspicion is required for diagnosis [2]. In most cases, the preoperative diagnosis is made on the basis of the presence of diaphragmatic elevation and herniation of the abdominal organs into the thorax [1, 4]. CT is the imaging modality of choice in assessment of both TDR and TAWH. In TDR, it not only confirms the diagnosis in suspicious or equivocal chest radiograph [2] but also can be used to assess the presence of associated intraabdominal injuries, which are reported in 70–90 % of patients [1]. In our patient, thoracoabdominal CT not only demonstrated TDR but also led to the diagnosis of TAWH and exclusion of other intraabdominal injury, leading to the successful undertaking of a laparoscopic approach.

TDR and TAWH rarely occur in isolation and are associated with other injuries, such as head, chest, abdominal, pelvic, and extremity injuries [3–7]. In TDR, thoracic aortic tear has also been reported [3]. These associated injuries, not the diaphragmatic tear, have accounted for early death in TDR [3, 4]. Therefore, clinicians treating patients with either TDR or TAWH should be alert for such a possibility, as delayed diagnosis may lead to untoward outcomes and even death.

Undoubtedly, surgical repair is the management of choice, as the defects in TDR and TAWH, no matter how small, will not heal spontaneously. However, the optimal timing of repair (prompt vs. delayed) has not been thoroughly investigated in the literature [5, 7]. While death and significant morbidity are rarely associated with delayed diagnosis [1, 4, 5, 7], there have been reports of complications such as bowel herniation, incarceration, and strangulation [3, 7, 13]. Therefore, early diagnosis is crucial to planning surgery to avoid potential complications. We recommend surgery at the time of trauma when the diagnosis is made. Primary repair of the diaphragm can be achieved in the acute setting because of the pliability of the diaphragm. Open approaches such as laparotomy and thoracotomy, or minimally invasive approaches such as laparoscopy and thoracoscopy, have been described. Nevertheless, most authors prefer a transabdominal approach because it allows assessment and treatment of primary and associated injuries.

Laparotomy is the common approach used in emergency treatment of ruptured diaphragm, and TDR has been shown to be successfully repaired in the majority of cases [3, 11]. However, over the last two decades, a minimally invasive approach has been added to the surgical armamentarium, and its benefits, such as early convalescence and reduced operative trauma, are well described [14, 15]. In our opinion, if the requisite surgical expertise is available, a laparoscopic approach should be considered first, especially in an obese patient with no other associated injuries. Diagnostic laparoscopy can exclude other intraabdominal injuries, thus avoiding unnecessary midline exploratory laparotomy and minimizing the procedure to local wound exploration and anatomic layered repair and thereby achieving the best long-term cosmesis [16]. However, laparotomy is the better option in the event of hemodynamic instability (as diaphragmatic tear is rarely the cause of significant bleeding) [1] and presence of concomitant intraabdominal injuries such as visceral organ or bowel injuries.

The concept of application of mesh suggests that, in the setting of high-velocity injuries with a substantial amount of tissue loss, use of a mesh should be avoided until the patient has demonstrated recovery. Mesh repair is not recommended for patients with associated solid organ or hollow viscus injury, as such injuries are sources of mesh infection. In a hostile environment with increased risk of infection, a biological mesh has been recommended as the alternative to synthetic mesh because of its inherent properties of being able to incorporate into the surrounding tissues with decreased risks of infection, adhesion, erosion, extrusion, and rejection compared with a synthetic mesh [17, 18]. However, there is a paucity of high-level evidence on which to base conclusive recommendations [17]. Our patient had extensive tissue loss in the musculoaponeurotic layers of the abdominal wall. Primary fascial repair will result in approximation under tension, leading to possible necrosis and a high rate of hernia recurrence. A primary repair with the use of synthetic mesh to bridge the fascial defect was our best option, as there was no hollow viscus injury. The placement of the mesh as an underlay graft behind the muscles with an overlap of at least 3 cm is most ideal, as it has been shown to be associate with a lower recurrence rate than onlay and inlay techniques [19]. A delayed repair after 6–8 weeks is another consideration. However, it is a formidable task that requires consideration of risks such as incarceration and strangulation of intraabdominal organs. It entails multiple surgeries, prolonged recovery, and additional morbidity as compared with a single-stage repair.

In our patient, there is a possibility of posttraumatic intercostal herniation of the lung in the future because there was a disruption of the thoracic wall by tearing of the intercostal muscles. We did not perform surgical repair of thoracic defect at the same setting, as it entails a separate thoracic intercostal incision. Because we have primarily closed the diaphragmatic defect and inserted a nonabsorbable mesh to reinforce the diaphragmatic and abdominal wall defect, we foresee that the recurrence rate and incidence of transdiaphragmatic intercostal hernia should be small. As a result, we opted for a “watchful waiting” approach to assess whether our patient will require a staged surgical repair for his thoracic wall defect later.

## Conclusions

We present a complex form of injuries that was managed successfully via a laparoscopic approach. Primary repair using mesh grafts in the setting of high-energy trauma is feasible but needs to be individually tailored. More importantly, it is imperative that such patients are expertly managed throughout their inpatient stay to identify any potential complications.

## Consent

Written informed consent was obtained from the patient for publication of this case report and any accompanying images. A copy of the written consent is available for review by the Editor-in-Chief of this journal.

## Abbreviations

AIS: Abbreviated Injury Scale
CT: computed tomography
ISS: Injury Severity Score
TAWH: traumatic abdominal wall hernia
TDR: traumatic diaphragmatic rupture
TRA: transversus abdominis muscle
US: ultrasound
